# Evidence of mycobacteriaemias and mycobacterial co-infections uncovered in cattle at slaughter using a novel phage-based PhMS-qPCR assay for viable *Mycobacterium bovis* and *Mycobacterium avium* subsp. *paratuberculosis*

**DOI:** 10.1186/s13620-025-00323-1

**Published:** 2025-11-29

**Authors:** Hannah Dane, Brendan Gilbride, Minu Thomas, Irene R. Grant

**Affiliations:** 1 Institute for Global Food Security, School of Biological Sciences, Queen’s University Belfast, Belfast, BT9 5DL Northern Ireland, UK; 2 Rapid-Myco Technologies Limited, c/o 19 Chlorine Gardens, Belfast, BT9 5DL Northern Ireland, UK

**Keywords:** Mycobacterium bovis, *Mycobacterium avium* subspecies *paratuberculosis* (MAP), Co-infection, Mycobacteraemia, Blood, Phagomagnetic separation (PhMS)-qPCR, Culture

## Abstract

Accurate diagnosis of Bovine Tuberculosis (bTB) in cattle is important for success of eradication programmes, but the current reliance on the single intradermal comparative cervical tuberculin (SICCT) test is not achieving disease eradication in all jurisdictions, including Northern Ireland (NI). In this study, a novel Phagomagnetic separation (PhMS)-qPCR assay to rapidly detect viable *Mycobacterium bovis* in bovine blood samples was evaluated. A total of 149 heart blood samples were collected from cattle at exsanguination point of the slaughter line at a NI abattoir between June and August 2023 – 74 from TB reactor cattle (compulsorily culled within days of a positive SICCT test result) and 75 from routine slaughter cattle (< 30 months). Peripheral blood mononuclear cells (PBMCs) were isolated and lysed to release any mycobacteria present before PhMS-qPCR and culture were performed to detect presence of viable *M. bovis*. The DNAs obtained were subsequently tested for evidence of viable *Mycobacterium avium* subsp. *paratuberculosis* (MAP) also. PhMS-qPCR results indicate that of the TB reactor cattle with conclusive PhMS-qPCR results, 7.5% of bloods tested positive for viable *M. bovis* only, 41.8% for viable MAP only and 28.4% showed evidence of co-infection (both *M. bovis* and MAP detected). Of the routine slaughter cattle with conclusive PhMS-qPCR results, 22.4% of bloods tested positive for viable *M. bovis* only, 19.4% for viable MAP only and 20.9% showed evidence of co-infection. Of the blood samples with conclusive culture results, 19.6% TB reactors and 25.4% routine slaughter cattle were *M. bovis* culture positive. No agreement was observed between the *M. bovis* PhMS-qPCR assay and *M. bovis* culture results (Kappa − 0.028, 95% CI -0.162 to 0.105). Results of this study provide proof-of-concept that the PhMS-qPCR assay is able to detect viable *M. bovis* in bovine blood, although a different qPCR assay with greater detection sensitivity will need to be identified going forward. A surprisingly high number of *M. bovis*/MAP co-infections were detected in the blood of NI cattle, which may be contributing to failure of the bTB eradication scheme. The latter warrants further investigation.

**Supplementary Information:**

The online version contains supplementary material available at 10.1186/s13620-025-00323-1.

## Introduction

*Mycobacterium bovis* is the causative agent of bovine tuberculosis (bTB), a disease that primarily affects cattle but may occur in other domesticated species, wildlife and humans [1]. The disease has a global distribution, with only a small number of countries considered bTB-free [1]. Control schemes have been successful in reducing bTB infection rates in some Western countries, with 18 of 28 European Union member states achieving officially bTB-free status (defined as herd prevalence of ≤ 0.1% annually for six consecutive years) by 2018 [2]. In Northern Ireland (NI), participation in a bTB eradication scheme for cattle has been compulsory since the late 1950 s, in an effort to improve the welfare and productivity of cattle and reduce the risk to public health via exposure to *M. bovis* infected animals or contaminated dairy products [2]. The scheme implements a test-and-cull policy, whereby all herds must be screened annually for bTB by the single intradermal comparative cervical tuberculin (SICCT) test, and SICCT test positive cattle are compulsorily culled and subjected to post-mortem examination when slaughtered. However, the NI bTB eradication scheme has been largely unsuccessful, and in recent years there has been a significant deterioration in the bTB situation according to a recent TB Strategic Partnership Group report [3]. Department of Agriculture, Environment and Rural Affairs (DAERA) bTB statistics show that as of February 2025, the herd and animal incidence rates for bTB are 10.76% and 1.156%, respectively [4]. The bTB herd prevalence has increased from 4.9% in 1997 to 14.69% in 2024 [3]. One criticism of the scheme has been its heavy reliance on SICCT test for bTB screening. This test is favoured because it is quick and may be carried out on farms; however, there are known issues with its reliability. Sensitivity of the assay has been estimated to be only 41–61% when applied to chronically infected herds in NI [5], meaning that a significant proportion of *M. bovis*-infected cattle may test false-negative by SICCT test. The poor sensitivity of the assay may be because it relies on detection of the immune response against *M. bovis* rather than detecting the pathogen directly, and the immune response to bTB may vary in different animals and at different stages of infection. False-negatives may be obtained in animals tested in the very early (pre-allergic) or advanced (anergy) stages of infection, those that are co-infected with other mycobacteria, or those that are immuno-suppressed due to drugs, stress or early post-partum [6]. The specificity of the SICCT test is higher in the NI context at 94–99% [5], but there is still a risk of false-positive results due to the high levels of similarity between *M. bovis* antigens and those of other mycobacterial species. For example, use of vaccines against *Mycobacterium avium* subspecies *paratuberculosis* (MAP), the causative agent of Johne’s Disease (JD), is limited in many countries due to known or possible interference with the SICCT test [7]. This means that cattle infected with mycobacteria other than *M. bovis* are potentially needlessly being culled, while cattle infected with *M. bovis* remain within herds acting as a reservoir of infection. The sensitivity and specificity of the SICCT and similar tests may also be affected by co-infections of *M. bovis* with other mycobacteria. The possibility of an animal being simultaneously infected with two or more pathogens is often overlooked during diagnosis. However, there is evidence that co-infections are very common from parasites and pathogens that cause chronic disease [8]. Co-infections of *M. bovis* and MAP have been reported in Chile [9], Spain [10] and Northern Ireland [11], with co-infection status affecting the sensitivity of the caudal fold tuberculin skin test [9] and MAP ELISA [10]. The effect of co-infections on test sensitivity is complex, as sensitivity may increase shortly after infection, but decrease later due to anergy [6].

Difficulty in diagnosing bTB, due to a lack of reliable detection methods, is a major hindrance to the success of eradication programmes. The ideal test would enable rapid, sensitive and specific detection of viable *M. bovis* (the infectious agent), rather than anti-*M. bovis* immune response, but no such tests are currently in routine use. The available tests can be divided into two groups: direct tests where the pathogen itself is detected, and indirect where the immune response against the pathogen is detected [1, 12]. Other than the SICCT test, there are two common types of indirect assay: cytokine release assays, and serological assays. However, as these tests rely on detection of the immune response against *M. bovis* rather than the infectious agent, they may have similar limitations to the SICCT test. The two most common direct detection methods are culture and polymerase chain reaction (PCR). Culture is the gold standard detection method because it is the only method by which viable *M. bovis* may be directly demonstrated [1]. However, it is too slow and labour-intensive to be used for routine screening, since *M. bovis* may take up to 8 weeks to grow to detectable levels [13]. PCR is used to detect DNA from *M. bovis* and is rapid and specific but is not able to differentiate between viable and non-viable pathogens.

There has been increasing interest in bacteriophage-based methods for detecting pathogenic mycobacteria in recent years [14, 15]. Bacteriophages are viruses that infect and kill host bacteria, and they may have a broad or narrow host range. They are relatively cheap and easy to produce, may be highly stable in the environment, and they are only able to replicate within viable host bacteria, making them an attractive option for bacterial detection [16]. Several phage-based detection methods have been developed for detecting mycobacteria of animal health significance, including the PMS-phage assay [17], the Actiphage^®^ Rapid assay [18] and the Phagomagnetic separation (PhMS)-qPCR assay developed at Queen’s University Belfast [19]. The latter assay is a patented test (EP4022095C0, pending US2023340617A1, WO2021058606A1) which employs a lytic mycobacteriophage bound to tosylactivated paramagnetic beads. During the PhMS-qPCR assay mycobacteriophage-coated beads are mixed with test samples and the mycobacteriophages bind to any mycobacteria present. The bead-cell complexes are then magnetically separated and washed twice, before being resuspended in a small volume of Middlebrook 7H9 broth and incubated to allow phage-induced lysis of viable mycobacteria to occur. Thus, in this test, mycobacteriophage-coated beads are used to capture and concentrate mycobacterial cells from samples, and then to subsequently lyse the viable mycobacterial cells to release host DNA to be detected by qPCR. The assay was originally developed and successfully used for the detection of viable *Mycobacterium avium* subsp. *paratuberculosis* (MAP) in bovine milk [20]. MAP is the cause of Johne’s disease (JD) which is another important mycobacterial disease with impacts on cattle health and productivity worldwide. The PhMS-qPCR assay has been demonstrated to be reproducible in other veterinary diagnostic laboratories and to have good detection (analytical) sensitivity and specificity for detecting viable MAP in bovine faeces and milk [21]. Since the mycobacteriophage involved is a broad-spectrum mycobacteriophage, the PhMS-qPCR assay has the potential to be used for the detection of other mycobacteria of animal or public health significance, including *M. bovis* and *Mycobacterium tuberculosis*, by combining PhMS with a qPCR assay specific for these alternative mycobacterial targets.

The primary aim of this study was to demonstrate that the PhMS-qPCR assay is applicable for detection of viable *M. bovis* in bovine blood samples, a sample type that is cleaner and arguably easier for veterinary practitioners to collect than faeces or milk. A second aim was to assess the detection rates of viable *M. bovis* in bloods of TB skin test reactor cattle (compulsorily culled within days of a positive SICCT test result) and routine slaughter cattle < 30 months old collected at a NI abattoir. Although not an original aim of the study, since the DNA sample obtained after PhMS and subsequent incubation will emanate from any viable mycobacterial cells present in the blood sample that the mycobacteriophage has been able to infect, we took the opportunity to test the extracted DNAs for presence of viable MAP also. In the NI context, both JD and bTB are prevalent and economically important production diseases of cattle, and the existence of co-infections and MAP infections interfering with the bTB SICCT results have been previously reported [11].

## Materials and methods

### Cattle blood collection

Between June and August 2023, six visits were made to the abattoir in Northern Ireland that slaughters all SICCT test positive (TB reactor) cattle that have had to be compulsorily condemned under national bTB legislation [22]. On each visit, 10–20 blood samples were collected at exsanguination point in the slaughter process from either consecutive SICCT test positive cattle (TB reactors) or consecutive routine slaughtered cattle < 30 months old over within a timeframe of ~ 1 h. The abattoir processes only TB reactor cattle on certain days of the week and only routinely slaughtered beef cattle < 30 months old (and some older dairy cows) on other days. Therefore, only bloods from either TB reactor or routine slaughter cattle were collected at each visit, not a mixture of the two types. The blood samples were heart bleed samples initially collected by an abattoir worker into 100 ml sterile pots, and then immediately transferred into 30 ml tubes containing sodium EDTA (approximately 2.2 mg/ml blood, Sigma-Aldrich, Poole, Dorset, UK) to prevent clotting. Kill number was recorded at point of blood sampling, so that additional test results could later be requested from DAERA once all PhMS-qPCR test results became available, but no identifying tag numbers were recorded. In total, 149 cattle were collected, 74 from TB reactors and 75 from routine slaughter cattle, emanating from ~ 20 and ~ 25 different herds, respectively.

### Isolation of peripheral blood mononuclear cells (PBMCs) and Lysis to release mycobacteria

Generally, blood testing commenced the morning after collection (within 24 h), and samples were kept at room temperature (20–21 °C) until processing commenced. Peripheral blood mononuclear cells (PBMCs) were isolated from each EDTA-treated blood sample. Four millilitres of blood was mixed with 40 ml Ammonium-Chloride-Potassium (ACK) lysing buffer (prepared in-house using 8.02 g ammonium chloride, 1 g potassium bicarbonate and 0.0372 g disodium EDTA per litre, all Sigma-Aldrich) and incubated at room temperature for 10 min. The samples were centrifuged at 300 x g for 5 min and the pellet was washed once in 10 ml PBS. After centrifugation at 300 x g for 5 min, the PBMC pellet was resuspended in 2 ml Middlebrook 7H9 broth supplemented with 10% (v/v) OADC (both Becton Dickinson) and 2 mM calcium chloride (Sigma-Aldrich) and incubated at room temperature for 15 min to allow osmotic shock to lyse the PBMCs and release internalised mycobacterial cells, before being vortexed thoroughly and divided into two aliquots. Half the PBMC sample (1 ml) was diluted with a further 1 ml 7H9/OADC/2 mM CaCl_2_ and processed by PhMS-qPCR, and the other half (1 ml) was split between solid and liquid culture media, as described below.

### PhMS–qPCR

Automated PhMS was performed on half of the resuspended PBMC fraction, essentially as described for milk [20] but with a couple of modifications: 2 ml of the resuspended PBMC sample were subjected to PhMS rather than 1 ml resuspended milk pellet, and a PurePrep 24D instrument and associated 5 ml plastic consumable strips (both Molgen Limited, London), rather than a Dynal Beadretriever (Kingfisher 1 mL) instrument and associated tube strips, was used. On each PhMS run, a PhMS-positive and PhMS-negative sample were included – the negative control consisted of 2 ml 7H9/10% OADC/2mM CaCl_2_ broth, while the positive control consisted of 2 ml broth spiked with 20 µl of a 4–5-week-old MAP culture (as viable *M. bovis* could not be used in Containment Level 2 laboratory). Fifteen µl of mycobacteriophage-coated beads (prepared in-house) were added to each PBMC and control sample. The PurePrep instrument mixed the blood samples with the beads for 30 min, washed the beads twice in 1 ml phosphate buffered saline containing 0.05% Tween 20 (PBST), and finally resuspended the beads in 50 µl Middlebrook 7H9/10% OADC/2 mM CaCl_2_ broth in an Eppendorf tube. The resuspended bead-cell complexes were immediately transferred to a 37 °C incubator for 3.5 h, before being placed in a heating block at 55 °C for 1 min and then placed on a magnetic rack for 2 min. The supernatant containing mycobacterial DNA was immediately transferred to a fresh tube and beads and cell debris were discarded. If time did not permit qPCR to be carried out on the same day, DNA samples were stored at −20 °C overnight, otherwise qPCR proceeded directly after completion of DNA harvesting. To determine if DNAs obtained from blood samples after PhMS required purification before qPCR (to remove potential residual PCR inhibitors from blood), half of the DNA sample (25 µl) was mixed with 75 µl DNA binding buffer and passed through a Zymoclean Clean and Concentrator column, following the manufacturer’s instructions (Zymo Research, California, USA). Samples were then tested by *M. bovis*- (and later MAP-) specific qPCR with and without Zymoclean purification.

A SYBR-Green qPCR targeting the RD4 region [23] was used for detecting *M. bovis* with some modifications. Each reaction was made up to a final volume of 20 µl and consisted of 10 µl PCRBIO SyGreen mix (PCR Biosystems, London, UK), 0.2 pmol/µl of forward primer CBS1 (5’-TTC CGA ATC CCT TGT GA-3’), 0.2 pmol/µl reverse primer CBS2 (5’-GGA GAG CGC CGT TGT A-3’) (both Invitrogen Life Technologies, Paisley, Scotland) and 5 µl of template DNA. PCR run conditions were as follows: polymerase activation at 95 °C for 3 min, 40 cycles at 95 °C for 5 s followed by 60 °C for 30 s, and then high-resolution melt curve analysis comprised of 95 °C for 15 s, and then temperature ramping from 55 °C to 95 °C. Two ECO qPCR thermocyclers were used for all qPCR assays (one Illumina, San Diego, California, USA, and the other PCRMax, Stone, Staffordshire, UK). Samples were considered *M. bovis* positive if a peak was observed corresponding to an amplicon (product) melting temperature (Tm) between 84.7 °C and 85.3 °C, based on the Tm range of the *M. bovis* positive controls. The positive control consisted of a pure *M. bovis* broth culture which had been enumerated by culture and heat-inactivated at 80 °C for 2 h before centrifugation at 12,000 x g to sediment cell debris. A 10-fold dilution series was then prepared in molecular grade water or TE buffer from stocks, and the 10^− 3^ dilution (~ 10^3^−10^4^ *M. bovis*/ml) was used for the PCR positive control. For the PCR negative control, molecular grade water (ThermoFisher Scientific) was used.

The DNA obtained from each PBMC sample after PhMS was also tested by a MAP-specific qPCR. The *IS900* Taqman qPCR assay was originally described by Sidoti et al. [24] and used as part of the PhMS-qPCR test for detection of MAP in milk with some modifications [20], including introduction of a double quenched ZEN probe to maximise detection sensitivity. Each reaction was made up to a final volume of 20 µl and consisted of 10 µl PCRBIO probe mix No-Rox (PCR Biosystems), 0.3 pmol/µl of forward primer IS900QF (5’-CCG GTA AGG CCG ACC ATT A-3’) and 0.3 pmol/µl reverse primer IS900QR (5’-ACC CGC TGC GAG AGC A-3’) (both Invitrogen Life Technologies, Paisley, UK), 0.25 pmol/µl probe IS900QP (5’-FAM/CAT GGT TAT/ZEN/TAA CGA CGA CGC GCA GC/Iowa Black FQ-3’, Integrated DNA Technologies, Iowa, USA) and 5 µl of template DNA. PCR run conditions were as follows: polymerase activation at 95 °C for 2–3 min, 40 cycles at 95 °C for 5 s followed by 60 °C for 30 s. A PCRMax ECO qPCR thermocycler was used for all IS900 qPCR assays. The positive control MAP DNA was prepared by standardising a 5 week-old MAP culture to OD_600nm_ 0.1 and preparing a 10-fold dilution series in 7H9/10% OADC/2mM CaCl_2_ broth. The dilutions were boiled at 95 °C for 25 min, and then the 10^− 3^ dilution (~ 10^3^−10^4^ MAP/ml) was used as a qPCR positive control. Molecular grade water was used for the qPCR negative control.

### Mycobacterial culture

The second half (1 ml) of each PBMC sample was cultured for *M. bovis* by inoculation of 500 µl into Middlebrook 7H9 broth supplemented with 10% (v/v) OADC (both Becton Dickinson and Company, Franklin Lakes, NJ, USA) and 500 µl onto a non-acidified pyruvate Lowenstein-Jensen (L-J) slope (E&O Laboratories Ltd, Bonnybridge, Scotland). Cultures were incubated for 8 weeks at 37 °C in the CL3 pathogen laboratory. Agar slopes were checked visually for evidence of colonies typical of *M. bovis*, while broth cultures were checked for growth by measuring turbidity (OD_600nm_) on a weekly basis. At the end of the incubation period, all broth samples were streaked onto Middlebrook 7H10/OADC agar to check if cultures were pure and to obtain single colonies. L-J slopes and 7H10/OADC agar plates with growth had one colony picked, mixed with 50 µl TE buffer (Sigma-Aldrich) and boiled at 100 °C for 25 min. DNA was also obtained directly from broth cultures by centrifugation of 1 ml culture at 12,000 x g for 10 min to obtain a cell pellet for DNA extraction. The cell pellet was resuspended in 700 µl TEN lysis buffer (2 mM EDTA, 400 mM NaCl, 10 mM Tris-HCl, pH 8.0, 0.6% SDS) containing 20 µg proteinase K (Sigma-Aldrich) and incubated overnight at 37 °C. The suspension was boiled at 100 °C for 25 min to kill any remaining viable *M. bovis*. Cells were then mechanically disrupted using a FastPrep-24™ instrument (Fisher Scientific, Loughborough, Leicestershire, UK) for 45 s at 6.5 m/s with 0.3 g of 0.1 mm zirconia/silica beads (Biospec Products, Bartlesville, OK, USA). DNA was extracted with phenol: chloroform: isoamyl alcohol 25:24:1, precipitated in isopropanol and resuspended in 50 µl Tris-EDTA (TE) buffer pH 8.0 (all Sigma-Aldrich). Resulting DNA was subjected to the *M. bovis-*specific SYBR-Green qPCR described above. If a PCR positive result was obtained (Tm 84.7–85.0 °C), that blood sample was deemed viable *M. bovis* positive. No MAP culture was performed during the main blood study because testing for viable MAP had not been an original objective of the study.

### Follow-up blood testing to check on aspects of blood collection, PhMS and culture

During March and April 2024, an additional 25 bloods from TB reactor cattle were collected from the same NI abattoir over two visits, for the purposes of checking that our blood collection, PhMS and culture protocols were optimal for viable *M. bovis* detection. One heart bleed sample was obtained per animal and immediately split into an EDTA-containing tube (2.2 mg/ml EDTA) and a heparin-containing tube (17 IU Lithium heparin/ml blood). Blood samples (4 ml) were processed using the PBMC separation method involving 40 ml ACK lysis buffer (as described above). The resulting 4 ml PBMC sample was then split into two equal parts so that half could be incubated for 3 h (potentially more optimal for *M. bovis* detection) after PhMS and the other half for 3.5 h, as used in the main blood study. An additional 2 ml of blood was processed separately by lysis in 20 ml ACK lysis buffer, washing with 5 ml PBS and resuspension in 1 ml broth for culture purposes. As in the main study, PBMCs were cultured for *M. bovis* on LJ pyruvate agar slopes. However, the liquid culture medium was changed to commercially available BD MGIT culture tubes (7 ml) supplemented with 10% MGIT OADC supplement and BD MGIT PANTA antibiotic supplement per manufacturer’s instructions (all Becton, Dickinson and Company). In addition, MGIT culture tubes supplemented with 10% MGIT OADC, MGIT PANTA and 2 µg/ml mycobactin J (Serosep Limited, Limerick, Ireland) were used to culture MAP (no solid culture medium for MAP was inoculated in parallel). Confirmation of isolation of either *M. bovis* or MAP was by PCR of suspect colonies or pellets from MGIT cultures. Both were boiled at 100 °C for 25 min in 50 µl TE buffer to inactivate viable *M. bovis* and extract DNA prior to qPCR.

### Statistical analysis of results

GraphPad Prism 10 (GraphPad Software, Boston, USA) was used for statistical analyses and preparation of figures. The online GraphPad Kappa calculator was used for calculation of Fleiss’ Kappa. A small number of samples had inconclusive *M. bovis* PhMS-qPCR results (those with a Tm within the correct range but with double adjacent peaks, or a Tm just outside the correct range). These were included as a third test result in Fleiss’ Kappa calculation but were excluded from graphs and the sample numbers adjusted accordingly.

## Results

### Information supplied by DAERA for cattle sampled

Table 1 summarises information supplied by the DAERA Veterinary Officer in relation to the 149 cattle whose blood was tested. The TB reactor cattle (*n* = 74) comprised of 31 beef animals and 43 dairy animals with mean ages at point of slaughter of 35 and 71 months, respectively. The mean mm difference (± standard deviation) recorded for the SICCT test for beef TB reactor animals was 12.1 ± 10.5 mm and for dairy TB reactor animals was 8.2 ± 8.1 mm (unpaired t-test *p* = 0.0732; see Additional File 1, Fig. 1). Around 40% of both types of TB reactor animal had shown visible lesions (VL) at post-mortem inspection, so ~ 60% had been recorded as non-visibly lesioned (NVL). Lymph nodes from just eight TB reactor cattle had been cultured for *M. bovis* (3 of 4 beef animals positive and 2 of 4 dairy animals positive) and only two TB reactor cattle had a gamma interferon test result (both positive). The routine slaughter cattle (*n* = 75) comprised of 71 beef animals < 30 months old and four dairy cows with mean ages at point of slaughter of 27 and 66 months, respectively. Only two of these cattle had shown visible lesions upon post-mortem inspection (LRS) and lymph nodes had been cultured for *M. bovis*, one of which was recorded as culture positive.

**Table 1:** Summary of information supplied by department of Agriculture, environment and rural affairs veterinary officer for the TB reactor (*n* = 74) and routine slaughter (*n* = 75) cattle whose blood was tested by the PhMS-qPCR assay and culture

Parameter	TB reactors			Routine slaughter			
	Beef (*n* = 31)	Dairy (*n* = 43)		Beef < 30 months (*n* = 71)		Dairy (*n* = 4)	
Mean age ± st. dev. (months)	35 ± 38.3	71 ± 34.3		27 ± 2.3		66 ± 34.0	
Mean SICCT result ± st. dev. (mm difference)	12.1 ± 10.5	8.2 ± 8.1		N/A		N/A	
Post-mortem inspection:							
No. (%) cattle with Visible lesions (VL)	13 (41.9%)	17 (39.5%)		N/A		N/A	
No. (%) cattle with Non-visible lesions (NVL)	18 (58.1%)	26 (60.5%)		N/A		N/A	
No. (%) cattle with Lesions at routine slaughter (LRS)	N/A	N/A		2/71 (2.8%)		0/4 (0%)	
Gamma interferon assay positive	None tested	2/2		N/A		N/A	
*M. bovis* culture positive	3/4	2/4		1/2		None tested	

N/A, not applicable

**Fig. 1:**
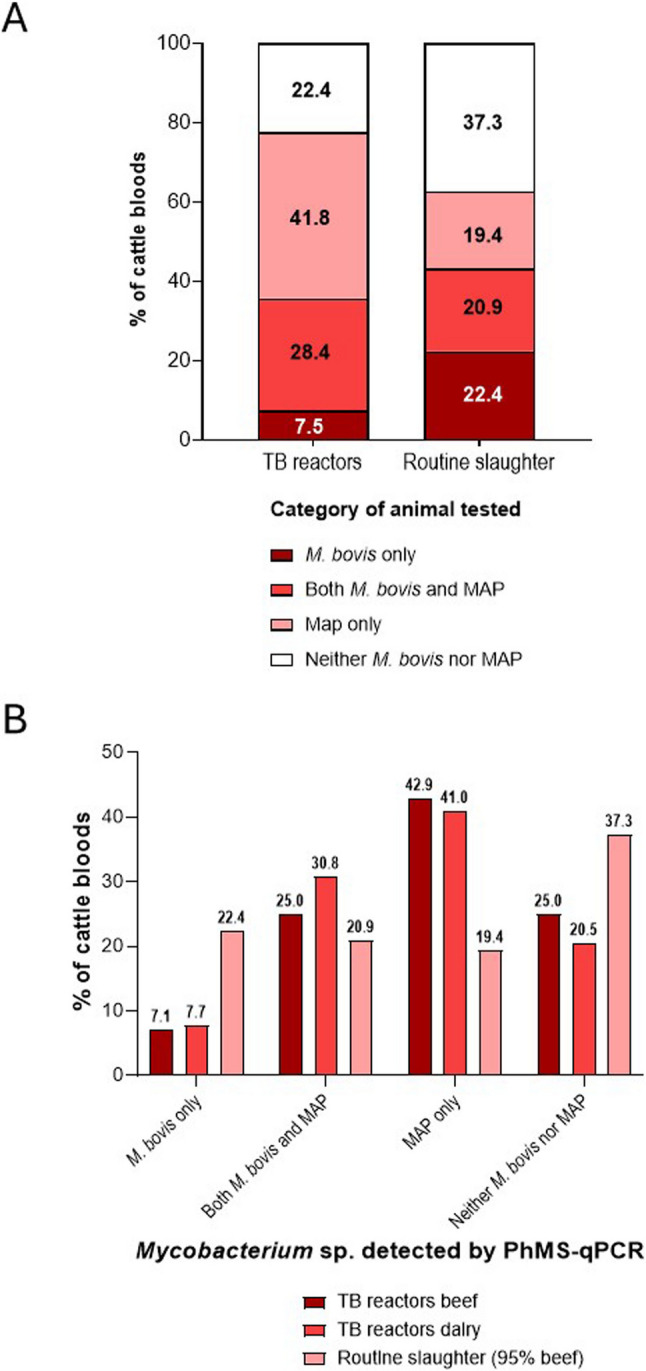
Breakdown of cattle blood PhMS-qPCR results showing MAP and/or *M. bovis* detections by **A** category of cattle tested (TB reactor or routine slaughter) and **B** by beef or dairy cattle (routine slaughter category was 95% beef cattle)

### Detection of viable *M. bovis* and viable MAP in blood by PhMS-qPCR

PhMS-qPCR results for both TB reactor and routine slaughter cattle are summarised in Fig. 1. Results for 15 cattle were excluded because they yielded inconclusive *M. bovis* PhMS-qPCR results (as defined above in Methods section). Among the TB reactors, 52 (77.6%) of 67 bloods tested positive for viable *M. bovis*, viable MAP or both mycobacterial pathogens, and so showed some form of mycobacteraemia. Among the routine slaughter animals, 42 (62.7%) of bloods tested positive for viable *M. bovis*, viable MAP or both mycobacterial pathogens. Numbers of MAP-positive animals were higher than the number of *M. bovis*-positive animals among TB reactors, but similar numbers of MAP- and *M. bovis*- positive animals were observed in the routine slaughter cohort. There were slightly more cattle in the routine slaughter cohort that were negative for both viable *M. bovis* and viable MAP (25 of 67 bloods, 37.3%) compared to the TB reactors (15 of 67 bloods, 22.4) (Fig. 1A). Surprisingly, there were more *M. bovis* positive PhMS-qPCR results for the routine slaughter cattle (29 of 67 bloods, 43.2%) compared to the TB reactor cattle (24 of 67 bloods, 35.8%). The biggest difference between the two groups was that there were almost twice as many MAP-positive results (either MAP only or MAP and *M. bovis* co-infection) for the TB reactor cattle (47 of 67 bloods, 70.1%) than for the routine slaughter cattle (27 of 67 bloods, 40.3%). A Fishers’s Exact test indicated that SICCT test results were significantly associated with *Mycobacterium* sp. or spp. detected (*p* = 0.0031); specifically, SICCT positive and *M. bovis* only *p* = 0.4799, SICCT positive and MAP only *p* = 0.0009, SICCT positive and co-infections of *M. bovis* and MAP (*p* = 0.4228) and SICCT positive and mycobacteraemia of any kind (*M bovis* only, MAP only or both species) *p* = 0.0887. The percentages of dairy and beef animals in each PhMS-qPCR result category for the TB reactor cattle were generally similar (Fig. 1B). Co-infections (*M. bovis* and MAP detected in the same blood sample) were frequently detected in the blood of both TB reactor and routine slaughter cattle (28.4% and 22.4% of animals, respectively). A Fisher’s Exact test indicated that cattle type (beef or dairy) was not significantly associated with *Mycobacterium* sp. or spp. detected (*p* = 0.1323).

**Fig. 2:**
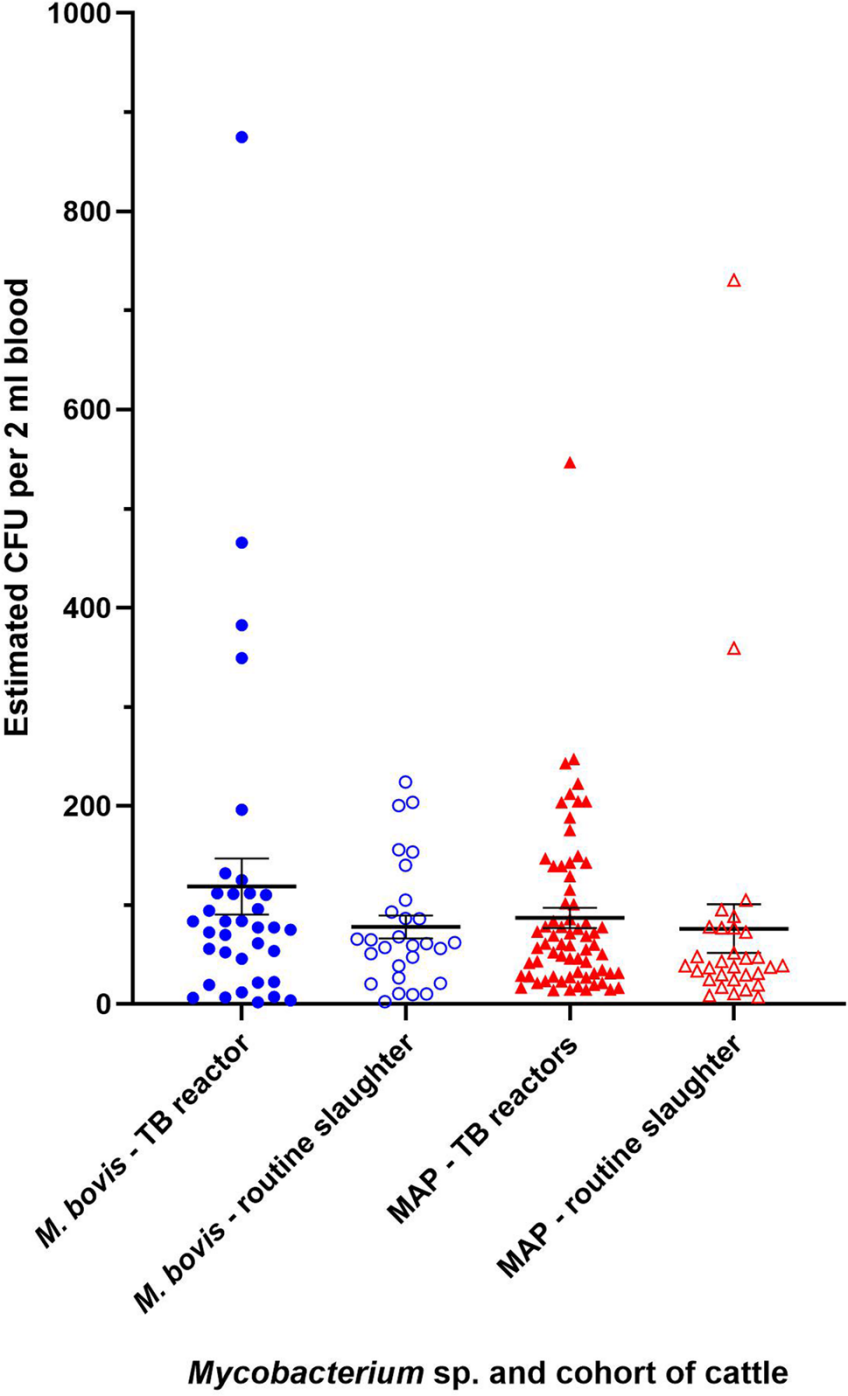
Estimated numbers of viable *M. bovis* and viable MAP detected in PhMS-qPCR positive blood samples from TB reactor and routine slaughter cattle. Error bars indicate mean ± standard error of the mean. Estimated CFU per 2 ml blood counts were calculated using equations of standard curves for the Kapalamula et al. [23] and Sidoti et al. [24] qPCRs employed. Unpaired t-tests indicate no significant difference between numbers of either pathogen detected in TB reactor cattle compared to routine slaughter cattle (*M. bovis* *p* = 0.2275, MAP *p* = 0.6322)

The PhMS-qPCR assay is not a truly quantitative assay, since 100% capture of mycobacteria from a test sample will never be achieved by PhMS. However, the Cqs the test generates can be used to provide an estimate of the number of viable *M. bovis* or viable MAP detected if related back to standard curves for the qPCRs employed. When this was done, the estimated number of viable *M. bovis* detected in cattle blood by PhMS-qPCR ranged from 2 to 875 CFU per 2 ml blood tested with a mean ± standard deviation of 112 ± 143 CFU per 2 ml blood tested, and the estimated number of viable MAP detected ranged from 7 to 731 CFU per 2 ml blood tested with a mean ± standard deviation of 70 ± 111 CFU per 2 ml blood tested (Fig. 2).

### Detection of viable *M. bovis* in blood by culture

*M. bovis* culture was attempted from PBMCs of 54 of the 74 TB reactors (the first 20 bloods were not cultured) and all 75 of the routine slaughter animals. Unfortunately, many of these cultures became overgrown with contaminating microorganisms, so the presence of *M. bovis* had to be identified by performing qPCR on pellets from broth cultures or colony sweeps from LJ slopes or colonies from 7H10/OADC agar sub-cultures. From these, 10 (19.6%) of 51 TB reactors and 16 (25.4%) of 63 routine slaughter cattle tested positive by culture for *M. bovis*. Three suspect cultures from TB reactors and 12 cultures from routine slaughter were excluded due to inconclusive qPCR results. MAP culture was not attempted from any of the blood samples in the main study.

**Fig. 3:**
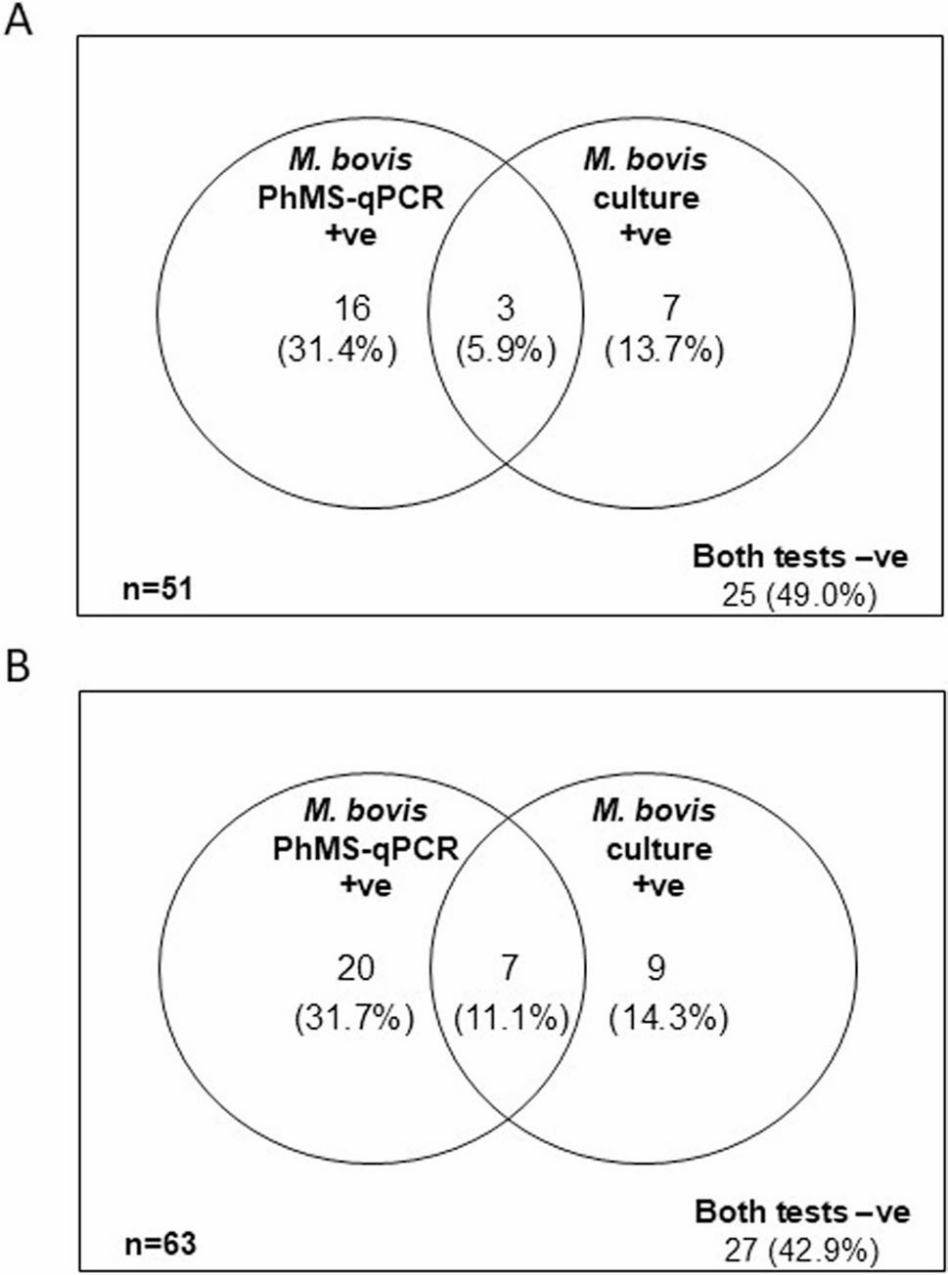
Venn diagrams showing interrelationships between PhMS-qPCR and culture results for detection of viable *M. bovis* in bloods of **A** TB reactors and **B** routine slaughter cattle

When *M. bovis* PhMS-qPCR results were compared with the culture results there was less agreement between the two methods than anticipated, with considerable numbers of samples testing positive by PhMS-qPCR only or culture only (Fig. 3). Overall, the Kappa score for *M. bovis* culture compared to *M. bovis* PhMS-qPCR was − 0.028 (95% confidence interval − 0.162 to 0.105) indicating no agreement between the two tests (see Additional File 1, Table 2).

### Follow-up checks on aspects of blood collection, PhMS and culture

**Fig. 4:**
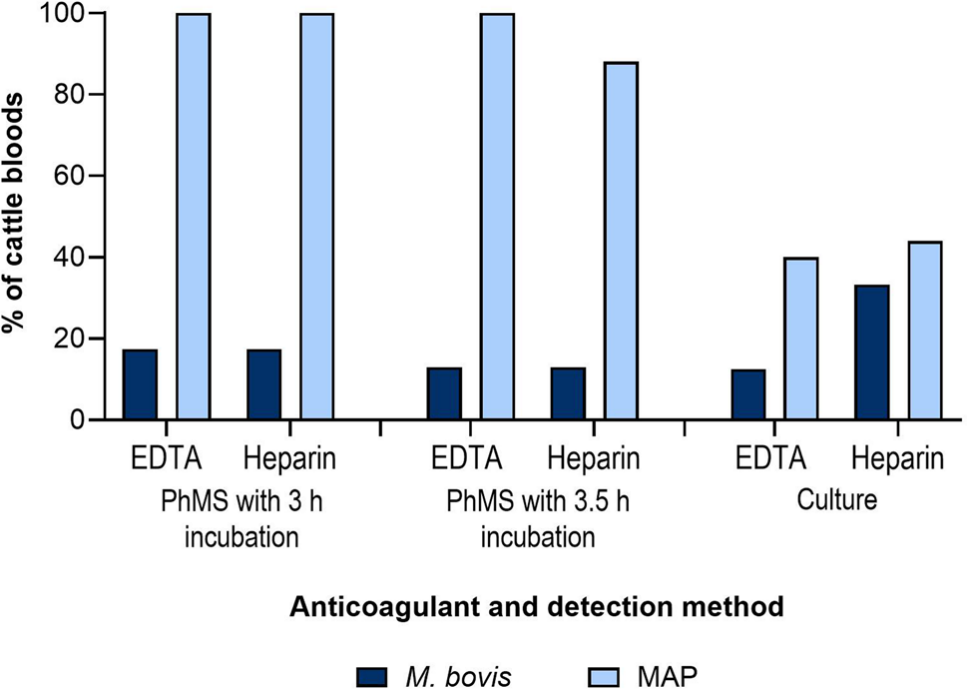
Effect of anticoagulant (EDTA or Heparin) used during blood collection on detection of viable *M. bovis* and viable MAPby PhMS-qPCR with 3 h or 3.5 h incubation post-PhMS and MGIT culture. Results relate to blood from 25 additional TB reactor cattle tested during follow-up checks

PhMS-qPCR results for the additional bloods from 25 TB reactors collected in March/April 2024 are shown in Fig. 4. No more than four samples were positive for *M. bovis* by any one of the sample collection, preparation and PhMS-qPCR method combinations studied, although in total 10/23 (43.5%) samples were positive by at least one PhMS-qPCR method (two samples had to be excluded due to inconclusive results). There were two more *M. bovis* PhMS-qPCR positive results after incubation for 3 h post-PhMS compared to 3.5 h (8 and 6, respectively). To our surprise, all 25 of the additional TB reactor blood samples tested positive for viable MAP via all of the sample collection, preparation and PhMS-qPCR method combinations studied, except for blood samples collected into heparin and incubated for 3.5 h after PhMS when 22 (88%) of 25 samples were positive (Fig. 4). Otherwise, there was no difference in PhMS-qPCR positivity between blood collection into EDTA or heparin when samples were incubated for 3 h after PhMS. The differences between the four variations of PhMS-qPCR were not significant as determined by the Friedman test (*P* = 0.5).

**Fig. 5:**
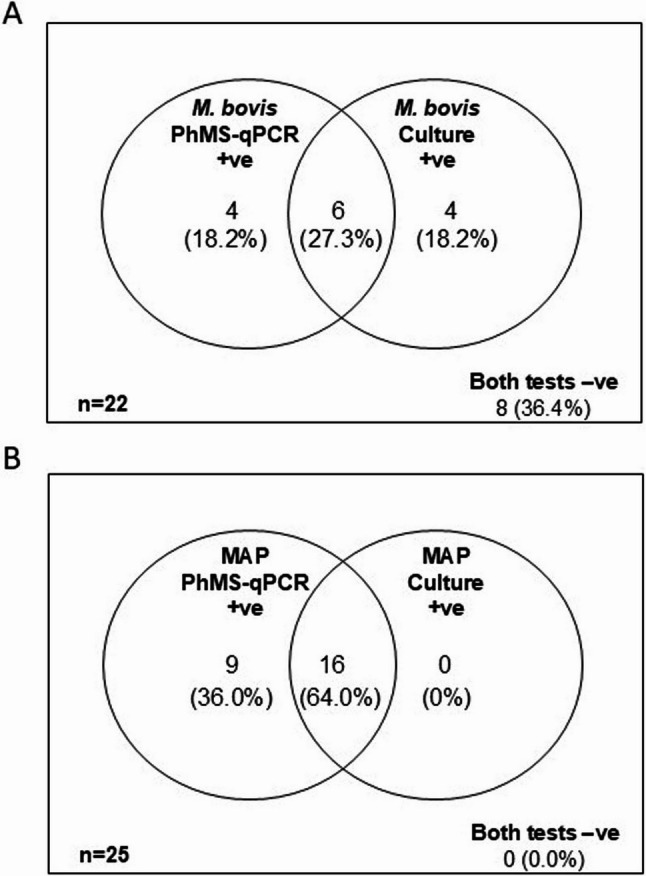
Venn diagrams showing interrelationships between PhMS-qPCR and culture results for the additional 25 bloods from TB reactor cattle tested to check on aspects of blood collection, PhMS, and culture. **A ***M. bovis* and **B** MAP

In total, 10 (41.7%) of the 25 additional cattle blood samples tested culture positive for *M. bovis* (one result inconclusive based on qPCR results of uncertain interpretation) and 16 (64%) of the 25 samples tested culture positive for MAP using the commercial MGIT culture tubes. In contrast to the *M. bovis* PhMS-qPCR results, there were more *M. bovis* culture positives from blood samples collected into heparin compared to those collected into EDTA (Fig. 4B), although this difference was not significant as determined by the Mann-Whitney test (*P* = 0.67). Interrelationships between PhMS-qPCR and culture results are visualised in Fig. 5. For *M. bovis*, there was slight agreement between PhMS-qPCR and culture results, with a Kappa agreement of 0.203 (95% confidence interval − 0.124 to 0.530). In contrast, for MAP there was no agreement between PhMS-qPCR and culture results (Kappa 0.000) for the additional 25 blood samples because no truly MAP negative samples had been tested.

## Discussion

The aims of this study were to apply a recently developed and optimised PhMS-qPCR assay for rapid detection of viable mycobacteria to test for viable *M. bovis* in cattle blood for the first time, and to ascertain the prevalence of *M. bovis* or MAP mycobacteraemia in cattle being slaughtered at a NI abattoir. Both *Mycobacterium* spp. were detected in the blood of TB reactors and cattle being routinely slaughtered, providing proof-of-concept that the PhMS-qPCR assay can detect viable mycobacteria in cattle blood samples. To our knowledge, this study is the first to provide both PhMS-qPCR and cultural evidence of the existence of mycobacteraemias in cattle, an understudied topic [25], and of co-infections of *M. bovis* and MAP, reinforcing previous reports of co-infections with MAP and *M. bovis* in cattle in NI [11].

Prior to this blood study commencing, the PhMS-qPCR assay had mainly been used for detection of MAP in bovine milk and faeces [20, 21], so preliminary experiments had been undertaken to optimise a sample preparation method for isolating PBMCs from cattle blood and identify a suitable qPCR method to use with PhMS for detection of *M. bovis* rather than MAP (data not presented). For blood collection, the impact of anticoagulants heparin and EDTA [26] on PhMS-qPCR results was considered and EDTA treated cattle bloods tested PhMS-qPCR positive for *M. bovis* most often. A range of different published PBMC separation methods was evaluated including: SepMate™−15 or SepMate™−50 PBMC isolation tubes (STEMCELL Technologies Ltd, Cambridge, UK) used with Lymphoprep™ (STEMCELL Technologies Ltd) or Ficoll Paque™ Plus density gradients (Cytiva, Cardiff, UK) [27, 28]; an ACK lysis method [29]; and a method utilising Roche Red Blood Cell lysis buffer (Roche, Basel, Switzerland) [30] (see Additional File 1 for further details). During this sample preparation optimisation phase, blood samples from cattle that were either SICCT test positive or had bTB confirmed based on other diagnostic tests (kindly provided by Colm Brady, Department of Agriculture, Food and Marine, Republic of Ireland) were tested. The ACK lysis method yielded the most *M. bovis* positive PhMS-qPCR results, so was deemed the best method to employ for the cattle blood study reported herein (see Additional File 1, Table 1). Several different *M. bovis*-specific molecular detection methods were evaluated for use in combination with PhMS for the blood study, including a Taqman qPCR assay [31], two SYBR-Green qPCR assays [23, 32] and three LAMP assays [33–35]. Although the Kapalamula et al. SYBR Green qPCR assay [23] was not demonstrated to be the most sensitive of the methods evaluated on DNA from a pure *M. bovis* culture, it proved to be the only method to yield any positive results when DNAs extracted from bovine blood samples by PhMS were tested. This may be because our Taqman probes use the reporter dye FAM, the emission wavelength of which has been reported to overlap with the absorption wavelength of blood [36]. Unfortunately, we were unable to source blood samples from confirmed bTB-free cattle to be able to demonstrate the resultant PhMS-qPCR assay’s diagnostic specificity, but the exclusivity of the selected Kapalamula et al. SYBR Green qPCR assay targeting the RD4 deletion in *M. bovis* [23] was demonstrated by testing DNA from pure cultures of 10 other *Mycobacterium* spp. available within the laboratory culture collection, none of which gave a positive result. Thus, based on results of these preliminary studies, during the main study all cattle bloods were collected into containers with EDTA present, PBMCs were isolated by the ACK lysis buffer method prior to PhMS, and DNA obtained after phage lysis of any mycobacteria captured from the sample by PhMS were subjected to the Kapalamula et al. qPCR [23].

Mycobacteraemia is rarely reported in cattle, but has previously been detected by culture [37, 38], PCR [37–39] or phage-based assays [18, 27]. Swift et al. [18, 27] reported detecting mycobacteraemia in a group of 41 SICCT-positive cattle using two phage-based detection methods. In the first study, 66% (27/41) of the cattle tested positive using a phage amplification assay, while in the second 95% (39/41) of the same cattle tested positive with the Actiphage^®^ Rapid assay. Consequently, the 35.8% PhMS-qPCR positivity for viable *M. bovis* in SICCT test positive cattle in this study is much lower than expected. The discrepancy may be due to differences between regions of the United Kingdom (England and Northern Ireland) and differences in *M. bovis* strains circulating in these regions, or the stage of disease in the animals studied, or may be due to differing levels of sensitivity and specificity of the three phage-based methods. The Swift et al. studies [18, 27] employed Recombinase Polymerase Amplification (RPA) and qPCR methods targeting the *IS6110* and *IS1081* insertion sequences, respectively, while the qPCR we selected to use for our blood study targeted the RD4 deletion [23]. The *IS6110* and *IS1081* insertion elements are present in multiple copies in members of the *M. tuberculosis* complex (which includes *M. bovis*), so provide a more sensitive target for detection of *M. bovis* compared to the RD4 deletion which is only found in *M. bovis*. It appears that the PhMS-qPCR assay may have underestimated the prevalence of *M. bovis* mycobacteraemia in NI cattle because a less-than-optimal qPCR in terms of analytical sensitivity was selected for the study. Further work is needed to identify a more appropriate *M. bovis* qPCR to combine with PhMS.

The number of routine slaughter animals that tested positive for *M. bovis* in their blood was higher than expected (43.2%). This is in contrast to Swift et al. [27] and Swift et al. [18], who reported that all 45 blood samples from SICCT test negative cattle they tested were negative by both the phage-RPA and Actiphage^®^ Rapid methods. It is not known when the SICCT tests had been administered to the routine slaughter cattle tested during our study, so they could have become infected after their last SICCT test. It is also possible that they could have tested false-negative with the SICCT test previously, which has limited sensitivity in herds with persistent bTB problems in NI [5]. The fact that one of the two lymph nodes cultured from the routine slaughter cattle with LRS was positive provides some evidence that cattle in the routine slaughter category cattle were not free from *M. bovis* infection.

The most surprising finding of the study was the high prevalence of MAP mycobacteraemia recorded, and particularly the number of co-infections with both *M. bovis* and MAP detected, in the blood of NI cattle at time of slaughter. Animals that were PhMS-qPCR positive for MAP and negative for *M. bovis* made up the biggest percentage of TB reactors (41.8%) followed by animals co-infected with both MAP and *M. bovis* (28.4%). Co-infected cattle were previously reported in NI by Byrne et al. [11], who found that cattle having a bTB breakdown were at increased risk of testing positive for MAP antibodies by a commercial ELISA assay. The percentage of cattle that were positive for MAP may have been higher than those positive for *M. bovis* in this study because the PhMS-qPCR assay was originally developed and optimised for MAP [20], and the *IS900* qPCR (multi-copy target) used for MAP detection has been demonstrated to have a lower detection limit in terms of CFU/ml than the RD4 qPCR (single copy target) used for *M. bovis* detection. Nevertheless, the high levels of MAP positivity among TB reactors suggests that SICCT interference by non-tuberculous mycobacterial infections or co-infections of *M. bovis* with another *Mycobacterium* sp. may potentially be playing a role in the failure of the bTB eradication scheme in NI.

Due to the low levels of *M. bovis* PhMS-qPCR positivity in TB reactors observed and the lack of agreement between PhMS-qPCR and culture results for *M. bovis* during the main blood study, follow up testing of an additional 25 blood samples obtained from TB reactor cattle at the same NI abattoir was undertaken. It was felt that several aspects of the methodology originally adopted for the main blood study needed to be checked. Firstly, if the initial choice of anticoagulant EDTA rather than heparin had been the correct one. Secondly, if an incubation time between PhMS and qPCR of 3 h rather than 3.5 h might have been more optimal for *M. bovis* detection (*M. bovis* burst time is 180 min, MAP burst time is 210 min; Grant, unpublished data). Thirdly, if culture of *M. bovis* in commercial BD MGIT medium plus associated supplements (BD MGIT OADC and BD MGIT PANTA antibiotics) might have yielded more culture positives than in-house prepared Middlebrook 7H9/OADC broth. From the results of the follow up testing, it appeared that blood collection into EDTA and incubation of samples at 37 °C for 3 h post-PhMS was optimal for detection of both viable *M. bovis* and viable MAP by PhMS-qPCR, whereas blood collection into heparin (which we did not use in the main blood study) may be more optimal for subsequent detection of *M. bovis* by culture. Ideally, more blood samples would need to be tested to confirm this finding. Given the biological similarities between MAP and *M. bovis* cells, it is likely that the optimal sample preparation, PBMC isolation and PhMS method for each species will be similar. We surmise that the poor detection sensitivity for *M. bovis* compared to MAP is most likely due to a less-than-optimal qPCR method having been employed for the former. As already mentioned above, further investigation will be required to identify a more sensitive but *M. bovis-*specific qPCR assay to combine with PhMS going forward.

The greatest limitation of this study is the lack of confirmation of PhMS-qPCR results by parallel conventional culture. Culture is the gold standard detection method for detecting viable *M. bovis* [1] and ideally would have been used for clarification of bTB status where there was disagreement between the PhMS-qPCR assay and the SICCT test results. We had hoped to validate the PhMS-qPCR results by comparison with statutory culture of lymph nodes (as had been done in a previous NI bTB study some years earlier, [40]. It was only when other laboratory results for the animals tested were returned by the DAERA veterinary officer that we became aware that statutory culture of VL and NVL lymph nodes from all SICCT test positive animals is no longer routinely performed. It transpired that a change in statutory bTB testing requirements in the intervening years meant more of a focus on herd level rather than individual animal level follow up investigation (personal communication, Dr Tom Ford, AFBI). Unfortunately, our attempts to culture *M. bovis* from the 149 cattle bloods on LJ pyruvate slants and in in-house prepared Middlebrook 7H9/10% OADC broth (without antibiotics added) were not very successful. Far too many cultures became overgrown quickly masking any *M. bovis* growth that may have occurred or preventing any *M. bovis* growth at all. Antibiotic supplement was not included in either the LJ medium or the 7H9/OADC broth we employed during the main blood study; we had wrongly assumed that this would be unnecessary for culture of blood which should be a comparatively clean sample. This likely explains why the switch to using commercial MGIT™ tubes with MGIT PANTA supplement added for the 25 extra blood samples tested increased the number of *M. bovis* culture positives from 19.6% to 40.0%; although the 40% *M. bovis* positivity rate is still lower than would be expected for TB reactor animals. *M. bovis* is typically cultured from lymph nodes or tissue samples rather than blood [41], and there are few reports of culture of *M. bovis* from bovine blood samples. Mycobacterial blood culture in ruminants is reported to have poor sensitivity [31, 42], with isolates typically only being obtained from animals with advanced disease, and presumably with a higher concentration of mycobacteria circulating in their blood. Ideally, for validation of a new *M. bovis* detection method, culture should be performed on both lymph nodes (to confirm infection status of the animal) and blood (to demonstrate the presence of mycobacteraemia) in parallel.

## Conclusion

A new mycobacteriophage-based PhMS-qPCR assay was applied to detect viable *M. bovis* and viable MAP in bovine blood samples for the first time. Both mycobacteria were successfully detected, providing proof of concept that this assay has sufficient analytical sensitivity for testing cattle bloods. More routine slaughter cattle blood samples than TB reactor cattle bloods tested PhMS-qPCR positive for *M. bovis*, so there was poor agreement between the phage-based assay and the SICCT test results. Further work is required to identify a more sensitive qPCR assay to pair with PhMS for detection of viable *M. bovis* to increase detection sensitivity. The second, very interesting, finding was that co-infections of *M. bovis* and MAP were surprisingly common in cattle being slaughtered in NI, accounting for 28.4% of TB reactor cattle and 19.4% of routine slaughter cattle in the sampled population. This finding provides important new evidence to support a previous hypothesis that co-infections may be playing a role in the failure of the current NI bTB control programme [11]. Further investigations and research in this area are certainly warranted.

## Supplementary Information

Supplementary Material 1

## Data Availability

The datasets used and analysed during this study, if not already available within the article and its supplementary information, may be requested from the corresponding author.
