# Supplementary material for: Evidence of mycobacteriaemias and mycobacterial co-infections uncovered in cattle at slaughter using a novel phage-based PhMS-qPCR assay for viable Mycobacterium bovis and Mycobacterium avium subsp. paratuberculosis
Source: Ir Vet J. 2025 Nov 29;79:2. doi: 10.1186/s13620-025-00323-1 (PMC12772088; doi:10.1186/s13620-025-00323-1)
Supplement: Supplementary file 1 — Supplementary Material 1 [file 13620_2025_323_MOESM1_ESM.docx]

**SUPPLEMENTARY INFORMATION**

**Evidence of mycobacteriaemias and mycobacterial co-infections uncovered in cattle at slaughter using a novel phage-based PhMS-qPCR assay for viable *Mycobacterium bovis* and *Mycobacterium avium* subsp. *paratuberculosis***

Hannah Dane^2^, Brendan Gilbride^2^, Minu Thomas^2^ and Irene R. Grant^1,2^*

^1^Institute for Global Food Security, School of Biological Sciences, Queen’s University Belfast, and ^2^Rapid-Myco Technologies Limited, both 19 Chlorine Gardens, Belfast BT9 5DL, Northern Ireland, United Kingdom

***Corresponding author:** Prof Irene R. Grant, [i.grant@qub.ac.uk](mailto:i.grant@qub.ac.uk)

**Details of the PBMC isolation methods evaluated prior to commencement of the blood study:**

**(1) SepMate™ tubes with Lymphoprep™ or Ficoll-Paque™ density gradients** *–* SepMate™-50 or -15 tubes were used as per the instructions of the manufacturer (STEMCELL Technologies Ltd, Cambridge, UK). The tubes were prepared by adding Lymphoprep™ Density Gradient (STEMCELL Technologies Ltd) to the tubes by pipetting through the centre hole. Whole blood preserved in Heparin- or EDTA-coated tubes was mixed with Phosphate-Buffered Saline at a ratio of 1:1. After this, each blood sample was poured down the side of the tube and allowed to mix with the density gradient. The samples were centrifuged at 800-1200 x g for 30 minutes at room temperature, with the brake on, to obtain separate layers. The top plasma layer was discarded, and the middle PBMC layer was transferred to a 15 ml centrifuge tube. The PBMC fraction was washed twice with 2 ml PBS, centrifuging at 300 x g for 8 minutes at room temperature, with the brake on, between washes. The PBMCs was resuspended in 5 ml 7H9/OADC/2 mM CaCl_2_ broth using a pastette before incubation at room temperature for 15 min to lyse them and release internalised mycobacteria. The Ficoll-Paque™ method was identical to the SepMate method except that the Lymphoprep™ was replaced with Ficoll Paque Plus (GE Healthcare UK Limited, Little Chalfont, UK).

**(2) ACK lysis method** *–* Ammonium-Chloride-Potassium (ACK) lysing buffer was prepared in-house as follows: 8.02 g ammonium chloride, 1 g potassium bicarbonate and 0.0372 g disodium EDTA (all Sigma-Aldrich) dissolved in a litre of distilled water and then autoclaved at 121^o^C for 15 min before use. Whole blood preserved in Heparin- or EDTA-coated tubes was mixed with ACK lysing buffer at a ratio of 1 part blood to 10 parts lysis buffer (2 ml and 20 ml or 4 ml and 40 ml). The samples were gently mixed by inversion and incubated at room temperature for 5, 10 and 15 min. The PBMCs were collected by centrifugation at 300 x g for 5 min, and the supernatant was discarded. The pellet was washed in PBS, centrifuged at 300 x g for 5 min, and the supernatant discarded. The PBMCs were resuspended in 2 or 4 ml 7H9/OADC/2 mM CaCl_2_ using a pastette before incubation at room temperature for 15 min to lyse them and release internalised mycobacteria.

**(3) Roche red blood cell lysis buffer** *-* Whole blood preserved in Heparin- or EDTA-coated tubes was mixed with Red Blood Cell Lysis Buffer (Roche, Basel, Switzerland) at a ratio of 1 part blood to 2 parts buffer (500 µl in 1 ml). The tubes were shaken for 10 min at room temperature. The tube was centrifuged at 500 x g for 5 min and the supernatant was discarded. The pellet was resuspended in Red Blood Cell Lysis Buffer and centrifuged at 500 x g for 3 min. The PBMCs were resuspended in 7H9/OADC/2 mM CaCl_2_ broth using a pastette before incubation at room temperature for 15 min to lyse them and release internalised mycobacteria.

PBMC samples generated by all methods then were processed through *M. bovis* PhMS-qPCR (see details in main text). Results in Table 1 show that the ACK lysing buffer method resulted in highest detection rates when cattle blood was collected into EDTA anti-coagulant.

**Table 1.** Summary of PhMS-qPCR results obtained after processing blood of confirmed TB cattle (DAFM, ROI) or TB reactor cattle (collected at NI abattoir) by different PBMC separation methods.

| **Expt no.** | **Source of blood samples** | **Anti-coagulant** | **No. of blood samples processed** | **Volume of blood tested (ml)** | **PBMC isolation method** | | | |
| --- | --- | --- | --- | --- | --- | --- | --- | --- |
|  |  |  |  |  | **Sepmate™-50 + Lymphoprep™** | **Sepmate™-15 + Lymphoprep™ or Ficoll Paque™ Plus** | **ACK lysis buffer** | **Roche RBC lysis buffer** |
|  |  |  |  |  | No. (%) samples PhMS-qPCR positive for viable *M. bovis* | | | |
| 1 | DAFM, ROI^¶^ | Heparin | 20 | 5 | 1 (5.0) | NT* | NT | NT |
| 2 | NI abattoir | Heparin | 10 | 5 | NT | 6 (60.0) | NT | NT |
|  |  |  |  |  |  |  |  |  |
| 3 | NI abattoir | Heparin | 10 | 5 | NT | NT | 6 (60.0) | NT |
|  |  |  |  |  |  |  |  |  |
| 4 | DAFM, ROI | EDTA | 10 | 2 | NT | 5 (50.0)** | 6 (60.0) | 4 (40.0) |
|  |  | Heparin | 10 | 2 | NT | 3 (30.0)** | 7 (70.0) | 5 (50.0) |
| 5 | NI abattoir | EDTA | 18 | 2 | NT | NT | **15 (83.3)***** | NT |
|  |  | Heparin | 18 | 2 | NT | NT | 9 (50.0)*** | NT |

^¶^ Blood samples from cattle with confirmed TB kindly provided by Colm Brady, Department of Agriculture, Food and Marine, Republic of Ireland.

*NT, not tested. ** Ficoll Paque™ Plus used instead of Lymphoprep™ as density gradient with SepMate-15 tubes. *** Overall positivity for *M. bovis* when DNA post-PhMS tested both with and without zymocleaning.

**Figure 1.** Distribution of SICCT test results for beef and dairy TB reactor cattle. Error bars indicate mean mm difference in size of PPB and PPA lumps ± standard error of the mean.


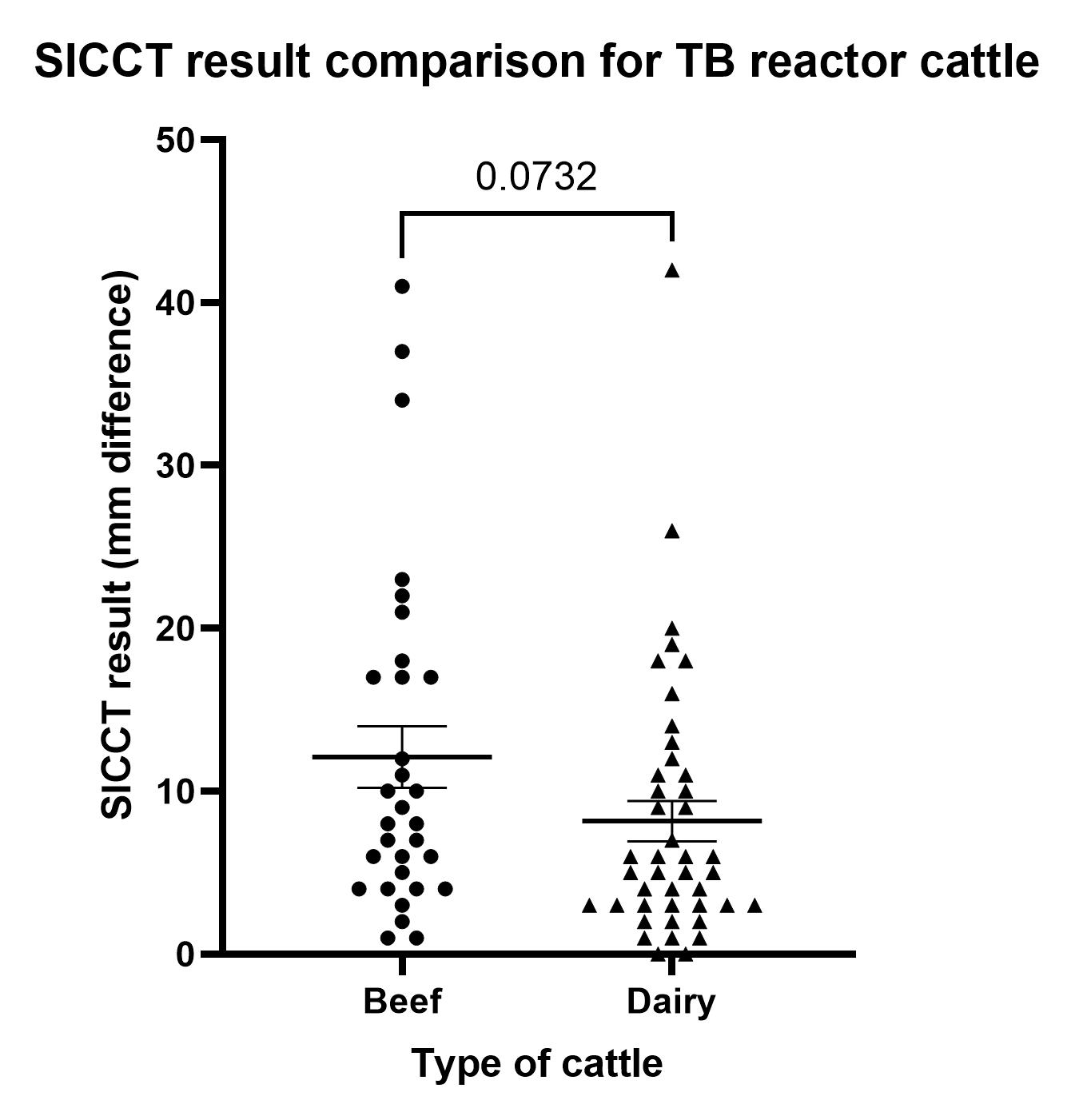


**Table 2.** 3x3 contingency table of *M. bovis* PhMS-qPCR and culture results for cattle blood samples used to calculate agreement between the tests.

|  | | **Culture (LJ slant or 7H9/OADC broth)** | | |  |
| --- | --- | --- | --- | --- | --- |
|  |  | **Positive** | **Inconclusive*** | **Negative** | **Total** |
| **PhMS-qPCR** | **Positive** | 10 | 2 | 36 | 48 |
|  | **Inconclusive*** | 1 | 0 | 8 | 9 |
|  | **Negative** | 16 | 4 | 53 | 73 |
| **Total** | | 27 | 6 | 97 | 130 |

* Inconclusive means that a very late Cq and Tm around correct value for *M. bovis* was observed for PhMS-qPCR or colony sweep or broth pellet qPCR, but melt curve result was difficult to interpret.

Number of observed agreements: 63 (48.46% of the observations)

Number of agreements expected by chance: 64.9 (49.89% of the observations)

**Kappa= -0.028**

SE of kappa = 0.068

95% confidence interval: From -0.162 to 0.105Kappa < 0: **No agreement**^[[1]](#footnote-1)^

1. Landis, J.R.; Koch, G.G. (1977). The measurement of observer agreement for categorical data. Biometrics. 33 (1): 159-174. doi: 10.2307%2F2529310 [↑](#footnote-ref-1)
